# Supplementary material for: North Carolina Macular Dystrophy: Phenotypic Variability and Computational Analysis of Disease-Associated Noncoding Variants
Source: Invest Ophthalmol Vis Sci. 2021 Jun 14;62(7):16. doi: 10.1167/iovs.62.7.16 (PMC8212441; doi:10.1167/iovs.62.7.16)
Supplement: Supplement 1 [file iovs-62-7-16_s001.pdf]

SUPPLEMENTARY MATERIAL

North Carolina macular dystrophy: phenotypic variability and computational analysis of disease-associated non-coding variants

David J. Green[1], Eva Lenassi[1-3], Cerys S. Manning[4], David McGaughey[5], Vinod Sharma[3], Graeme C. Black[1,2], Jamie M. Ellingford[1], Panagiotis I. Sergouniotis[1-3,6]

[1] Division of Evolution and Genomic Sciences, School of Biological Sciences, Faculty of Biology, Medicines and Health, University of Manchester, UK.

[2] Manchester Centre for Genomic Medicine, St Mary's Hospital, Manchester University NHS Foundation Trust, Manchester, UK.

[3] Manchester Royal Eye Hospital, Manchester University NHS Foundation Trust, Manchester, UK.

[4] Division of Developmental Biology and Medicine, School of Medical Sciences, Faculty of Biology, Medicines and Health, University of Manchester, UK.

[5] Ophthalmic Genetics and Visual Function Branch, National Eye Institute, National Institutes of Health, Bethesda, Maryland, United States.

[6] Institute of Biochemistry and Molecular Genetics, Faculty of Medicine, University of Ljubljana, Slovenia.

*Corresponding Author: Panagiotis I. Sergouniotis FRCOphth, PhD; Manchester Centre for Genomic Medicine, Saint Mary's Hospital, Oxford Road, Manchester M13 9WL, UK; email: panagiotis.sergouniotis@manchester.ac.uk.

Supplementary Table 1: Selected heterozygous single-nucleotide variants identified in the 560 kilobase (kb) region around the chr6:100,040,987G>C variant (corresponding to an identity-by-descent segment shared between families 75898 and 89794)*

**chromosome**	**position** (GRCh37 /hg19)	**dbSNP rsID**	**ref**	**alt**	**zygosity** (case 75898.2)	**zygosity** (case 89794.1)	**gnomAD allele count**	**gnomAD allele frequency**
chr6	100431554	rs7773011	T	C	0/1	0/1	11027	0.352
chr6	100431644	rs7749425	C	T	0/1	0/1	11218	0.358
chr6	100432655	rs7754794	C	T	0/1	0/1	11387	0.364
chr6	99948376	rs75064329	A	T	0/1	0/1	193	0.006
chr6	99956872	rs72926237	G	A	0/1	0/1	1106	0.035
chr6	99992605	rs72926287	G	A	0/1	0/1	1036	0.040

* Two study participants, 75898.2 and 89794.1 underwent genome sequencing. Sequencing data for patient 75898.2 were generated using the Complete Genomics platform and pipeline (v2.5) (see Ellingford *et al.*[15] for further information). Sequencing in patient 89794.1 was performed by Edinburgh Genomics using the Illumina TruSeq Nano DNA kit and the Illumina MiSeq platform; a minimal median coverage of 30-fold was achieved and variants were assessed and filtered using the Variant Annotation and Filter Tool (VarAFT) (see de Bruijn *et al.*[27] for further information).

After variant calling, DNA sequence alterations in the genomic region around the chr6:100,040,987G>C (GRCh37/hg19) variant were evaluated in the two probands. The maximum presumed identity-by-descent segment around this mutation was then determined through the detection of discrepant homozygous calls between the probands; a 560 kb region, chr6:99867067-100433110 (GRCh37/hg19), was highlighted. Within this region we identified 219 variants that were both shared between the families and present in the Genome Aggregation Database (gnomAD). These included multiple heterozygous variants that had a minor allele frequency <5%; some of these changes are shown above. These observations suggest that cases 75898.2 and 89794.1 share a rare haplotype (minor allele frequency <5%) that flanks the chr6:100,040,987G>C variant.

ref, reference sequence; alt, alternative sequence; gnomAD, Genome Aggregation Database v3.1

Supplementary Table 2: Top 10 predicted *PRDM13*-linked enhancers detected using the Activity-by-Contact (ABC) method with chromatin accessibility data from human macular tissue as input

chr	start	end	activity	distance	HiC contact	ABC Score
chr6	100036361	100036861	12.645519	18038	0.024038	0.010803
chr6	100046330	100046830	3.361263	8069	0.056099	0.006515
chr6	100038137	100039060	7.103467	16050	0.024038	0.006068
chr6	100037142	100037642	6.886428	17257	0.024038	0.005883
chr6	100049572	100050072	2.322015	4827	0.056099	0.004501
chr6	100039139	100039639	3.774571	15260	0.024038	0.003225
chr6	100040653	100041153	2.974766	13746	0.026677	0.002807
chr6	100043983	100044483	2.138283	10416	0.026677	0.002017
chr6	100066458	100066958	4.254949	12059	0.009265	0.00151
chr6	100093765	100094265	2.213217	39366	0.013686	0.001116

Supplementary Table 3: Top 10 predicted *PRDM13*-linked enhancers detected using the Activity-by-Contact (ABC) method with chromatin accessibility data from human retinal tissue as input

chr	start	end	activity	distance	HiC contact	ABC Score
chr6	100036361	100036861	19.248011	18038	0.024038	0.018074
chr6	100038619	100039588	14.615735	15545	0.024038	0.013724
chr6	100046348	100046848	5.313465	8051	0.056099	0.01132
chr6	100037098	100037598	9.831378	17301	0.024038	0.009232
chr6	100093380	100094216	4.319864	39149	0.013686	0.002395
chr6	100066419	100066919	4.759981	12020	0.009265	0.001856
chr6	100091066	100091566	1.609914	36667	0.013686	0.000892
chr6	100072214	100072714	2.321279	17815	0.007601	0.000762
chr6	100027633	100028516	4.246895	26574	0.003256	0.000708
chr6	100089821	100090321	1.099151	35422	0.013686	0.000609

Supplementary Table 3. Expression levels of *PRDM13* as identified in single-cell RNA-seq studies of the human retina

Cell type	No. cells detected	Total cells	% of cells with detectable expression	Expression log2(CPM+1)
Amacrine cell / horizontal cell precursors	934	1891	49.39	5.23
Amacrine cells	10184	118913	8.56	0.88
Horizontal cells	2132	27388	7.78	0.79
Neurogenic cells	322	12978	2.48	0.25
Photoreceptor precursors	157	9887	1.59	0.16
Pericytes	77	9345	0.82	0.08
Endothelial	170	22138	0.77	0.07
Retinal precursor cells	285	37465	0.76	0.07
Astrocytes	23	3403	0.68	0.06
Bipolar cells	808	147915	0.55	0.06

CPM, counts per million. All values were obtained from the https://plae.nei.nih.gov/ resource (accessed 12/2020).

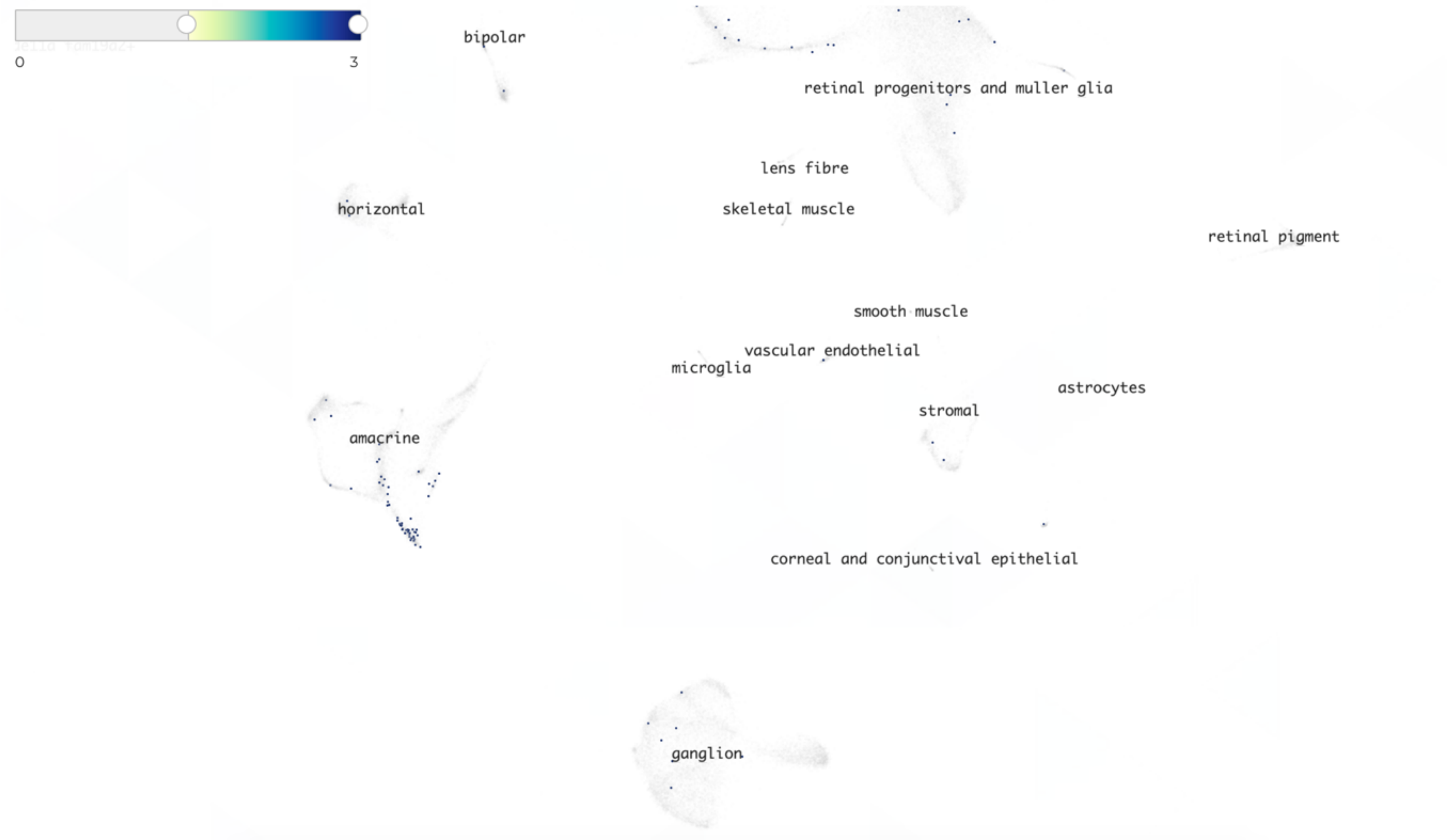

Supplementary Figure 1: UMAP (Uniform Manifold Approximation and Projection) plot showing single-cell expression of *PRDM13* in different cell types. Expression ranges from low (yellow) to high (blue). Cells with low expression have been hidden by setting the cut-off at 0.5. Data from https://descartes.brotmanbaty.org/ (accessed 12/2020).
